# Role of ammonia in NAFLD: An unusual suspect

**DOI:** 10.1016/j.jhepr.2023.100780

**Published:** 2023-04-25

**Authors:** Karen Louise Thomsen, Peter Lykke Eriksen, Annarein JC. Kerbert, Francesco De Chiara, Rajiv Jalan, Hendrik Vilstrup

**Affiliations:** 1 Department of Hepatology and Gastroenterology, Aarhus University Hospital, Denmark; 2 UCL Institute of Liver and Digestive Health, University College London, United Kingdom; 3 European Foundation for the Study of Chronic Liver Failure, Barcelona, Spain

**Keywords:** Ammonia, Cellular senescence, Epigenetics, Glutamine metabolism, Non-alcoholic fatty liver disease, Urea synthesis

## Abstract

Mechanistically, the symptomatology and disease progression of non-alcoholic fatty liver disease (NAFLD) remain poorly understood, which makes therapeutic progress difficult. In this review, we focus on the potential importance of decreased urea cycle activity as a pathogenic mechanism. Urea synthesis is an exclusive hepatic function and is the body’s only on-demand and definitive pathway to remove toxic ammonia. The compromised urea cycle activity in NAFLD is likely caused by epigenetic damage to urea cycle enzyme genes and increased hepatocyte senescence. When the urea cycle is dysfunctional, ammonia accumulates in liver tissue and blood, as has been demonstrated in both animal models and patients with NAFLD. The problem may be augmented by parallel changes in the glutamine/glutamate system. In the liver, the accumulation of ammonia leads to inflammation, stellate cell activation and fibrogenesis, which is partially reversible. This may be an important mechanism for the transition of bland steatosis to steatohepatitis and further to cirrhosis and hepatocellular carcinoma. Systemic hyperammonaemia has widespread negative effects on other organs. Best known are the cerebral consequences that manifest as cognitive disturbances, which are prevalent in patients with NAFLD. Furthermore, high ammonia levels induce a negative muscle protein balance leading to sarcopenia, compromised immune function and increased risk of liver cancer. There is currently no rational way to reverse reduced urea cycle activity but there are promising animal and human reports of ammonia-lowering strategies correcting several of the mentioned untoward aspects of NAFLD. In conclusion, the ability of ammonia-lowering strategies to control the symptoms and prevent the progression of NAFLD should be explored in clinical trials.

**Key points**

• The mechanisms underlying the multiorgan symptoms and transition of NAFLD from bland steatosis to steatohepatitis, cirrhosis and hepatocellular carcinoma are incompletely understood.
• Urea cycle activity is compromised in NAFLD, likely owing to epigenetic changes and increased hepatocyte senescence causing hyperammonaemia.
• Hyperammonaemia has widespread negative effects, contributing to liver fibrosis, sarcopenia, compromised immune responses, increased oncogenicity and cognitive disturbances.
• We suggest that the ammonia accumulation that results from decreased urea cycle activity contributes to the symptoms and progression of NAFLD.
• Ammonia lowering may be a novel therapeutic strategy to control the symptoms and prevent the progression of NAFLD.

## Introduction

Non-alcoholic fatty liver disease (NAFLD) afflicts about 25% of the global population, closely related to the prevalence of adiposity.^1^ NAFLD is often considered the hepatic manifestation of the metabolic syndrome, wherein lipids accumulate in ectopic sites such as the liver. The fatty liver disease in itself is associated with cerebral, muscular and several other extrahepatic manifestations.^2^^,^^3^ The disease is generally a health threat due to its association with cardiovascular disease and other extrahepatic diseases.^4^ The liver-related impact of the disease increases when it progresses from bland steatosis to non-alcoholic steatohepatitis (NASH) and fibrosis, to cirrhosis with its well-known serious complications, and hepatocellular carcinoma.^5^ The chronic exposure of the liver to free fatty acids, exceeding its capacity for normal energy metabolism by β-oxidation leads to lipotoxicity, triggering pro-inflammatory and pro-apoptotic signalling pathways and inducing mitochondrial, peroxisomal and microsomal fatty acid oxidation, with a resulting increase in the formation of hepatocyte-damaging reactive oxygen species.[6], [7], [8] The on-going liver inflammation also induces activation of hepatic stellate cells (HSCs), leading to collagen deposition and the development of liver fibrosis.^9^ However, it remains unclear how NAFLD leads to multiorgan symptoms and which factors cause the transition from supposedly innocuous hepatocyte fat infiltration to the dangerous stages of the liver disease. Such knowledge is of obvious importance given the huge number of persons at risk. Therefore, there is an avid and continuous quest for possible mechanistic elements explaining the symptomatology of NAFLD and its progression. Despite the high level of research activity, the field remains wide open.

One obvious way to look for such mechanisms is to focus on the functional consequences of hepatocyte fat infiltration. It has been proposed that fatty liver exhibits reduced liver regeneration capacity after partial hepatectomy, although data are conflicting[10], [11], [12] and changes in single mitochondrial and microsomal hepatocyte systems have been described.[13], [14], [15] Still, it has been the general understanding that fatty liver has no severe functional consequences. However, recent research shows that the condition does indeed compromise several metabolic liver functions, even in the early stages of NAFLD before advanced fibrosis and cirrhosis occur.[16], [17], [18], [19], [20] The synthesis of urea is one such liver function that is affected by steatosis. This function is particularly pathophysiologically relevant because it is present exclusively in hepatocytes and it is essential to the maintenance of life. Intact urea synthesis and its appropriate regulation are a prerequisite for normal whole body nitrogen homeostasis and hence for maintenance of normal body composition and health.

**Fig. 1:**
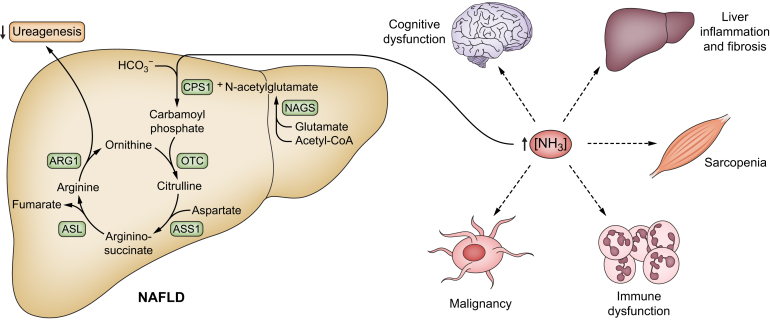
Hyperammonaemia may explain multiorgan symptoms and disease progression in NAFLD. ARG1, arginase 1; ASL, argininosuccinate lyase; ASS1, argininosuccinate synthetase 1; CPS1, carbamoyl phosphate synthetase 1; NAFLD, non-alcoholic fatty liver disease; NAGS; N-Acetylglutamate synthase; NH_3_, ammonia; OTC, ornithine transcarbamylase.

One of the important and exclusive functions of urea synthesis is the on-demand and definitive disposal of ammonia. Compromised urea synthesis invariably results in hyperammonaemia. We propose that ammonia is of importance for the symptomatology and progression of NAFLD. The underlying mechanism of this reduction in urea cycle activity is thought to be due to the effect of hepatocyte fat, which in itself damages the genetic regulation of urea synthesis, leading first to ammonia accumulation in the liver at the cellular level, and eventually to systemic hyperammonaemia.^19^ Derangements in glutamine metabolism might also play a role, with increased glutaminolysis resulting in ammonia production and further demands for urea synthesis.^21^^,^^22^ Liver ammonia accumulation has been shown to initiate and maintain a hepatic inflammatory response that may trigger the transition of bland steatosis to NASH and further towards cirrhosis.^23^ Systemic hyperammonaemia is toxic to several organs and the problem is aggravated with the progression of NAFLD, with further dysfunction of the urea cycle and liver zonation affecting glutamine synthetase. Thus, ammonia may be an unexpected pathogenic factor, contributing to multiorgan symptoms and disease progression in NAFLD, Fig. 1. In this review we present the case for ammonia as a pathogenic factor in NAFLD, go through the evidence implicating urea synthesis and other contributory mechanisms, and give hints at how the problem may be further studied and addressed.

## Urea synthesis and its regulation

Urea cycle activity is an exclusive hepatic metabolic liver function. It is phylogenetically old and has served a variety of functions during the evolution of species,^24^ from osmotic filler to metabolic elimination of excess bicarbonate.^25^^,^^26^ In mammals, urea synthesis serves in whole body nitrogen homeostasis through its central role in amino acid metabolism. When amino-nitrogen is available in excess, it is eliminated from the body via the synthesis of urea^27^^,^^28^ so that the regulation of urea production is the key to whole-body nitrogen balance. Urea synthesis is an irreversible process as mammalian cells have no urease activity.^29^

**Fig. 2:**
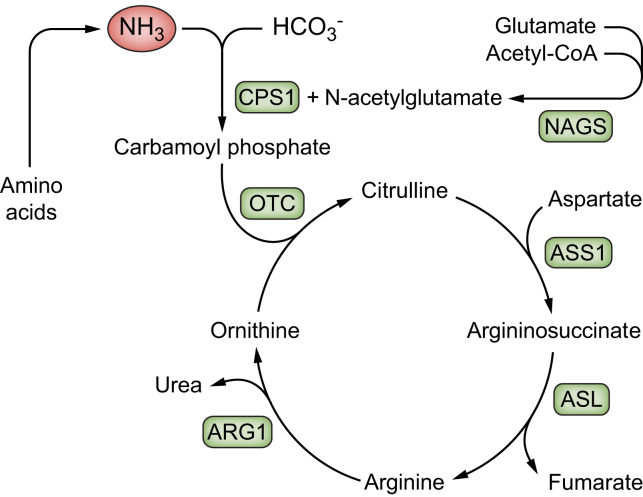
Urea cycle and its enzymes. ARG1, arginase 1; ASL, argininosuccinate lyase; ASS1, argininosuccinate synthetase 1; CPS1, carbamoyl phosphate synthetase 1; HCO_3_-, bicarbonate; NAGS; N-Acetylglutamate synthase; NH_3_, ammonia; OTC, ornithine transcarbamylase.

The five steps in the urea cycle are catalysed by five enzymes. The first and second cycle enzymes, viz. carbamoyl phosphate synthetase 1 (CPS1) and ornithine transcarbamylase (OTC), are mitochondrial, while the other cycle enzymes, viz. argininosuccinate synthetase 1, argininosuccinate lyase, and arginase 1, the last finally producing urea,^30^ are cytosolic (Fig. 2)*.* As the urea cycle is located partially in the mitochondria and partially in the cytoplasm, its steps are not completely stoichiometrically coupled. This means that urea cycle reactants are produced in excess and are released into the blood when the cycle is loaded. For instance, ornithine, which is not present in proteins, is needed for the urea cycle to run and is produced by the cycle itself.^31^ The cycle rate is controlled by the activity of the cycle feeder enzyme CPS1. The moment-to-moment activity of CPS1 is determined by the concentration of its obligatory allosteric activator N-acetyl-glutamate and its long-term regulation by induction of the transcription of the *CPS1* gene. In health, the capacity of the cycle is so abundant that near-saturation is never approached.^28^

The rate of urea synthesis is linearly and immediately dependent on its physiological substrate, the blood concentration of α-amino nitrogen, even in patients with cirrhosis ^28^; that said, non-substrate regulation of urea synthesis also takes place via modifications to this relationship.^32^ The intake and composition of food are important regulators; a high protein intake gradually upregulates urea synthesis capacity via cycle enzyme induction, whereas glucose intake rapidly decreases it.[33], [34], [35] Also, various hormones regulate urea synthesis in different directions. The strongest and most important upregulator is glucagon, which is involved in moment-to-moment regulation via its effects on N-acetyl-glutamate and in long-term regulation via induction of the urea cycle genes.[36], [37], [38] Downregulation of glucagon by glucose and its insulin response is responsible for the fall in urea synthesis and body nitrogen loss following carbohydrate ingestion, an effect lost in cirrhosis.^34^^,^^35^ Like glucagon, cortisol and adrenaline increase urea synthesis,[39], [40], [41] whereas growth hormone and insulin-like growth factor-1 both downregulate it.^42^^,^^43^ Finally, appropriate functional liver mass is essential for adequate urea production, and it follows that urea synthesis capacity is decreased in patients with cirrhosis or compromised liver function.^28^^,^^44^

Free amino acids in the blood pool are continuously and rapidly utilised, converted, and metabolised – they are used for protein synthesis and when metabolised their carbon skeletons are used for processes such as gluconeogenesis or fatty acid synthesis. Ammonia is produced when amino acids are available in excess of protein synthesis and their carbon skeletons are metabolised, leaving behind ammonia. Ammonia production by intestinal microbiota also contributes. As ammonia is highly toxic to several tissues, several mechanisms are available to remove it. The first line mechanism is transamination into non-essential amino acids. However, this is not an option when relevant carbon skeletons are not available or when amino acids are already abundant. The next option is amidation of glutamate to glutamine, which carries two nitrogen atoms, by the enzyme glutamine synthetase. This ammonia scavenging mechanism is ubiquitously active, including at a pivotal location in the perivenous hepatocytes, and has a very high affinity for ammonia. However, its enzyme capacity is small and limited by the availability of glutamate, and it does not definitively dispose of ammonia from the body. So, in the end, the body depends on urea synthesis to eliminate excess amino-nitrogen originating from surplus amino acids.

## Dysregulation of urea synthesis in NAFLD

Several lines of investigation suggest an association between NAFLD, impairment of urea synthesis, symptoms of NAFLD, and progression of liver injury and fibrosis. As early as the 1990s, it was shown that long-chain fatty acids promote perturbations in urea cycle enzyme gene expression, resulting in hyperammonaemia in rat primary cultured hepatocytes.^45^ A few years later it was demonstrated that hepatic triglyceride accumulation in cows inhibits ureagenesis and increases plasma ammonia concentrations.^46^ Experiments from our own group in 2014 pursued these findings in an animal model. In rats fed a high-fat high-cholesterol diet to induce NAFLD, we found a reduction in gene expression of urea cycle enzymes, in particular OTC, and showed that this resulted in downregulation of *in vivo* urea synthesis capacity.^18^ Using the same model, we found a progressive reduction over time in the expression and activity of urea cycle enzymes, resulting in hyperammonaemia and fibrosis progression.^47^ These changes were reversible upon recovery from NAFLD.^19^ Also in methionine- and choline-deficient mice with fatty liver, urea cycle enzyme genes were found to be downregulated^48^ and similar findings, alongside hyperammonaemia, were observed in *foz/foz* mice fed a high-fat diet.^49^

*In vitro*, the findings were confirmed in primary steatotic hepatocytes, demonstrating decreased gene expression of urea cycle enzymes and increased ammonia levels in the supernatant, alongside increased gene expression of pro-fibrogenic markers. Also, in precision cut liver slices, we demonstrated increased gene expression of pro-fibrogenic markers following lipid and/or ammonia exposure.^47^

Additionally, also human NAFLD is associated with a reduction in gene and protein expression and activity of urea cycle enzymes, as well as impairment of urea synthesis resulting in hyperammonaemia.^17^^,^^19^^,^^20^^,^^47^ In patients with steatosis and NASH, a progressive decrease in OTC enzyme concentration and activity was observed, which was associated with increased plasma and hepatic ammonia concentrations.^19^ Moreover, we observed a functional reduction in the *in vivo* capacity for ureagenesis and, at the same time, downregulation in the gene expression of most urea cycle-related enzymes, especially in patients with steatosis.^17^^,^^20^

## Other potential contributing mechanisms

**Fig. 3:**
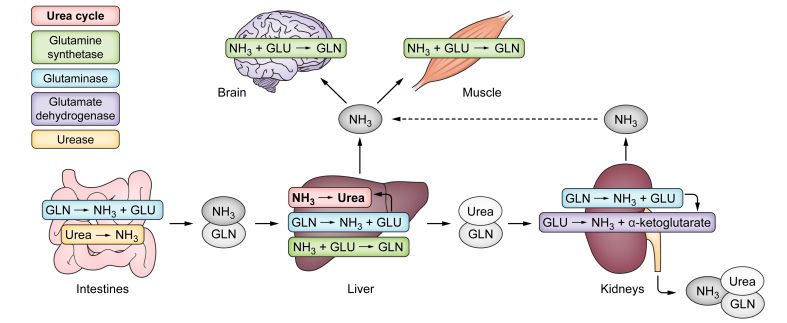
Main metabolic pathways and enzymes for ammonia production and clearance. GLN, glutamine; GLU, glutamate; NH_3_, ammonia.

Although the urea cycle in the liver is without question the key player in ammonia metabolism and elimination, other pathways and organs are also involved, Fig. 3. Glutamine metabolism is important and takes place both in the perivenous hepatocytes in the liver and in many other organs. The gut, kidneys and muscles all play a role in ammonia production, detoxification and excretion (involving glutamine metabolism and other mechanisms). In cirrhosis, secondary organ system dysfunction can lead to diminished ammonia detoxification and increased production, but its contribution to hyperammonaemia in NAFLD has not been adequately investigated and is currently unknown.

### Hepatic glutamine metabolism

Emerging evidence suggests a disruption of glutamine metabolism in NAFLD. In 1988, Kaiser *et al.* observed that glutamine synthesis was decreased *in vitro* in steatotic liver slices exposed to ammonium,^50^ whereas *in vivo*, increased hepatic gene and protein levels of glutamine synthetase have been observed in various animal models of NAFLD.^21^^,^^22^^,^^49^ In patients with NAFLD, one study found a reduction in hepatic glutamine synthetase that correlated with disease severity,^19^ whereas another study found an increase at the gene level.^20^ Also, glutaminolysis seems to be increased in NAFLD. Recent studies have reported overexpression of hepatic glutaminase 1 in both mouse models of diet-induced NASH and in the livers of patients with NASH.^21^ In both animal and human NAFLD studies, the glutamate/glutamine ratio levels were found to be increased in the liver and blood, reflecting increased glutaminolysis.^22^^,^^51^ However, the studies failed to show^21^ or report^22^ accumulation of ammonia in the liver or hyperammonaemia, indicating that disturbances in the glutamine/glutamate system alone are probably not enough to increase ammonia levels.

### Other organs

About half of the circulating ammonia load is derived from the gut. Bacterial urease-hydrolysis of urea in faeces water, metabolism of dietary proteins and the enzymatic breakdown of amino acids (particularly de-amidation of glutamine) result in the formation of ammonia, which subsequently diffuses into the portal circulation. Glutamine is the major energy source for small intestinal enterocytes and therefore, enterocytes have high glutaminase activity and low glutamine synthetase activity, further contributing to intestinal ammonia release.[52], [53], [54] Duodenal glutaminase activity has been reported to be four-fold higher in patients with cirrhosis compared to healthy controls, probably contributing to their hyperammonaemia^55^ together with deficient urea synthesis. In NAFLD, however, one animal study found no change in the gene expression of duodenal glutaminase 1,^49^ but this has not been investigated in human NAFLD. However, microbiota alterations potentially lead to an over-abundance of urease-producing bacteria in patients with NAFLD.^56^^,^^57^

Glutamine synthetase activity is relatively low in muscles,^58^ yet they can greatly impact on ammonia metabolism due to their great surface and mass. Ammonia can be both taken up and released by the muscles,^59^ but ammonia uptake is limited in healthy individuals.^60^ However, in patients with cirrhosis, high arterial ammonia levels appear to drive net muscle ammonia uptake and lead to net glutamine production.^59^ In line with this, patients with cirrhosis and sarcopenia have been found to be more likely to present with hyperammonaemia^61^^,^^62^ and, as NASH and obesity are associated with a high risk of sarcopenia,^63^ the same might be the case in these patients.

Renal ammonia production and excretion are essential for maintenance of acid-base homeostasis and are regulated by a variety of factors involving extracellular pH, potassium and several hormones. Renal ammoniagenesis predominantly results from glutamine metabolism by kidney-type glutaminase. In the physiological state, besides urinary ammonia excretion, there is a net ammonia release from the kidneys into the renal vein.[64], [65], [66] In patients with cirrhosis and hyperammonaemia, this blood release decreases significantly,^67^ which seems to act as a protective mechanism in early hyperammonaemia^68^ and the kidneys are even able to further increase urinary ammonia excretion.

So far, it has been consistently shown that urea synthesis is disturbed in human and experimental NAFLD, but there is circumstantial evidence that other mechanisms play a part in increasing ammonia levels in these patients. As urea synthesis is the only on-demand high-capacity system for elimination of ammonia, the decreased urea cycle capacity in NAFLD will play a central role, notwithstanding other disturbances in ammonia metabolism.

## Potential mechanisms of urea cycle dysfunction in NAFLD

The operative mechanisms for the observed damage to urea cycle genes and enzymes and impairment of physiological urea synthesis in NAFLD have not been fully elucidated, but epigenetic changes and cellular senescence are candidates.

### Epigenetic changes

We have shown that DNA hypermethylation of the promoter regions of urea cycle enzyme genes are involved,^19^ as detailed below. Epigenetic mechanisms are associated with the development and progression of NAFLD and may mediate the effects of environmental factors such as a western-style diet.[69], [70], [71], [72] Thus, epigenetic alterations have been found to be involved in the regulation of lipid metabolism, mitochondrial damage, oxidative stress and inflammation.^69^ Both in experimental fatty liver disease and in human NAFLD, altered DNA methylation of genes involved in steatohepatitis and the development of fibrosis was observed and these changes were more pronounced in more severe disease, again suggesting a mechanistic role for DNA methylation in the progression of NAFLD.[73], [74], [75], [76], [77] These changes were reversible after weight loss and bariatric surgery, which led to remodelling of the epigenetic signature.^78^^,^^79^

Specifically, in nitrogen metabolism, hypermethylated transcription-repressed genes involved in ureagenesis and amino acid metabolism have been observed in human NAFLD.^79^ Furthermore, downregulation of the flux-generating urea cycle enzyme CPS1 in patients with NASH was demonstrated by proteomic analyses.^80^ Also, our own *in vitro* and *in vivo* experimental and human studies suggested that hypermethylation of the promoter regions of urea cycle enzyme genes is one of the regulatory mechanisms responsible for the observed changes in urea cycle enzymes induced by steatosis. We found that accumulation of lipids in primary rat hepatocytes induces hypermethylation of the *OTC* promotor gene and eventually a decrease in the gene expression of *OTC*. These observations were extended by *in vivo* studies demonstrating hypermethylation of the promoter region of *OTC* in rats and of both the *CPS1* and *OTC* genes in patients with NAFLD, which in fact overlapped with hypermethylated regions in the *OTC* gene in rats.^19^ However, the mechanism by which hepatocyte fat accumulation results in increased methylation, predominantly of the mitochondrial urea cycle enzyme genes, has not yet been clarified.

### Cellular senescence

Cellular senescence refers to a state of stable cell cycle arrest that can be triggered by various types of cellular and environmental stress. Cellular senescence is suggested to play a role in modulating inflammation and the accumulation of fat in NAFLD.^81^ An important indicator for the presence of senescence in NAFLD is the overexpression of the tumour-suppressor gene p53 in human biopsies, which is a canonical inducer of senescence.^82^ The few studies exploring the relationship between cellular senescence and hyperammonaemia suggest that ammonia may directly induce senescence in astrocytes and hepatocytes.[83], [84], [85], [86], [87] This includes overexpression of p53, that has been found to suppress ureagenesis via transcriptional downregulation of urea cycle enzymes.^88^ Along similar lines, p53 activation by ammonia in mice led to a reduction in the expression of urea cycle genes.^88^ Conversely, knock-down of urea cycle genes activated p53, suggesting a bidirectional relationship between senescence and urea synthesis. Considering the increased expression of p53 in NAFLD animal models and human liver tissue, senescence may contribute to the further suppression of urea cycle function in patients with NAFLD and thus make them prone to hyperammonaemia, but this mechanism has not been fully explored.

## Ammonia: more than a neurotoxin

**Fig. 4:**
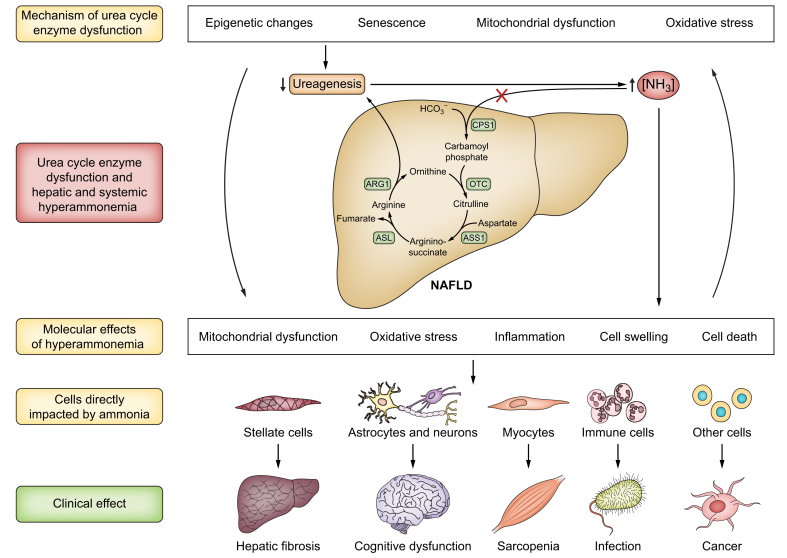
The causes and deleterious effects of hyperammonaemia in NAFLD. ARG1, arginase 1; ASL, argininosuccinate lyase; ASS1, argininosuccinate synthetase 1; CPS1, carbamoyl phosphate synthetase 1; NAFLD, non-alcoholic fatty liver disease; NH_3_, ammonia; OTC, ornithine transcarbamylase.

A decrease in the capacity for ureagenesis, as seen in cirrhosis and NAFLD, compromises the patient’s ability to eliminate ammonia, classically resulting in an increased risk of dyscognition and eventually overt hepatic encephalopathy (HE). However, other organs are also affected, Fig. 4.

### Cognitive dysfunction

The brain is the most studied organ in relation to hyperammonaemia due to the serious clinical problem of HE in liver disease. High ammonia levels in combination with systemic inflammation play central roles in the pathogenesis of HE.^89^^,^^90^ Humans and animals with NAFLD suffer from cerebral functional deficits. In recent years, cognitive deficits have been increasingly recognised as a complication of NAFLD, including in the early stages of the disease when there is no evidence of liver failure.^91^ The patients often have problems with memory, attention, concentration, forgetfulness and confusion, which is associated with a negative impact on everyday living and quality of life.[92], [93], [94] Most recently, a comprehensive study on neuropsychological functions in patients with NAFLD confirmed impairment in attention, mental concentration, psychomotor speed, cognitive flexibility, inhibitory mental control, and working memory.^95^ Such deficits have clear parallels to minimal HE, but it remains uncertain whether high ammonia is causally and obligatorily involved in the same way. Likewise, the term for this mental condition of NAFLD remains unsettled. HE is not appropriate because this term by definition assumes the presence of advanced liver disease.

### Induction of liver fibrosis

In the liver, hyperammonaemia is an important driver of activation of HSCs.^23^ HSCs are the main cell type responsible for extracellular matrix deposition and are key in the development of fibrosis and portal hypertension.^96^ We have previously demonstrated that pathological ammonia concentrations produce changes in the behaviour of cultured human HSCs, including significant alterations in cellular morphology, reactive oxygen species production and further HSC activation.^23^ Removal of ammonia from the cell cultures restored HSC morphology and function towards normality, indicating that the changes in HSCs induced by ammonia are reversible.^23^ These *in vitro* data were substantiated *in vivo* in bile duct-ligated rats with advanced fibrosis and hyperammonaemia where pharmacological ammonia lowering reduced HSC activation and portal pressure.^23^

### Sarcopenia

Sarcopenia and NAFLD are associated conditions.^3^^,^[97], [98], [99], [100] The relationship becomes more prominent with progression of disease, as seen in patients with NASH and fibrosis, and is independent of metabolic risk factors.[98], [99], [100] In NASH cirrhosis, sarcopenia associates with increased mortality as in other cirrhosis aetiologies.^63^

Hyperammonaemia resulting from compromised urea synthesis may constitute a mechanistic link between NAFLD and sarcopenia, similar to that proposed for cirrhosis.^101^ Pathological levels of ammonia may induce metabolic and molecular changes with detrimental effects on muscle mass and function.^101^^,^^102^ It has been suggested that high ammonia levels not only favour muscle formation of glutamine from glutamate, but also the conversion of α-ketoglutarate to glutamate.[103], [104], [105] This leads to depletion of tricarboxylic acid cycle intermediates, which in turn impairs ATP generation. This relative energy failure causes impaired contractile function together with increased muscle protein break down and reduced protein synthesis.^103^^,^^105^ Hyperammonaemia-induced alterations in mitochondrial metabolism also induces increased generation of reactive oxygen species coupled with oxidative damage to muscle protein.^105^ Moreover, ammonia itself may contribute to the development and aggravation of sarcopenia by increasing myostatin expression, a known inhibitor of protein synthesis and activator of autophagy.^104^^,^[106], [107], [108] Conversely, sarcopenia could cause hyperammonaemia due to the reduced detoxification capacity of glutamine synthetase.^109^ Accordingly, ammonia may be a driver as well as a result of sarcopenia. Also, it should be noted that sarcopenia is not merely a question of mass, but also of muscle quality and strength. Indeed, a recent study reported intact and even increased muscle mass in patients with NAFLD, but these patients also exhibited myosteatosis.^110^ In cirrhosis, myosteatosis has been associated with increased blood ammonia levels and risk of encephalopathy.^111^

### Immune dysfunction

Patients with NAFLD have increased mortality from infections,^112^ however, the mechanisms behind this observation have not been well investigated. Previously, we observed a dysfunctional innate immune response following endotoxin exposure in rats with diet-induced NASH and reduced urea synthesis.^113^ The functionality of circulating immune cells is also compromised in human NASH^114^ and we found that patients with NAFLD demonstrated increased activation and functional priming of blood neutrophils, which was most marked in patients with NASH.^115^ We suggest that hyperammonaemia contributes to the immune dysfunction characteristic of NAFLD in the same way as proposed in patients with cirrhosis.^116^ Ammonia may reduce neutrophil chemotaxis^117^ and induce neutrophil swelling and impaired phagocytosis.^116^ Also, hyperammonaemia reduces dendritic cell count, antigen uptake and allogenic lymphocyte stimulation through cell swelling, excessive reactive oxygen species production and mitochondrial dysfunction in dendritic cells from mice, as well as diminishing dendritic cell phagocytosis *ex vivo* in samples from mice and patients with cirrhosis.^118^

### Cancer

NAFLD is associated with an increased risk of cancer in general^119^ and particularly of hepatocellular carcinoma even before the development of cirrhosis.^120^ The increased cancer risk may be related to hyperammonaemia. Regarding the high risk of hepatocellular carcinoma, the structural changes associated with NAFLD, including hypo-vascularisation, favour the growth of cells that use ammonia as a nitrogen source for DNA synthesis, and ammonia increases the proliferation rate of cancer cells.^121^ It was already demonstrated in 1955 that immortalised adenocarcinoma cells increase their growth rate in the presence of ammonia, which acts as a nitrogen donor for the *de novo* biosynthesis of pyrimidine.^122^ Patients with metastatic cancer exhibit markedly greater ammonia accumulation in the liver compared to patients with mild and severe liver disease.^123^ The addition of ammonia to cell culture media has been shown to increase proliferation of breast cancer cells,^124^ while loss of urea cycle enzymes in some tumour cells promotes proliferation by facilitating pyrimidine synthesis.^125^ It has also been demonstrated that ammonia promotes proliferation of cancer cells lacking the tumour suppressor p53.^88^ Further in support of the role of ammonia in hepatocellular carcinoma, the anti-cancer effect of targeting the high expression of heat shock proteins in such tumour cells seems to inhibit the use of ammonia by cancer cells for malignant transformation.^126^ There is thus evidence indicating that ammonia is involved in oncogenesis, and particularly hepatocarcinogenesis.

## Limitations of published data

Taken together, there is a large body of data incriminating urea synthesis and ammonia in NAFLD. However, the data have to be sought for in many different categories of publications often not focused directly on the issue, and none of them cover the complete chain of evidence. Interpretative extrapolations are necessary to piece together the full picture. The most direct data and the largest experimental data mass rely on animal NAFLD models with the limitations implied by this approach. In humans there are solid data on the impairment of urea synthesis but less so on hyperammonaemia, and only associative data on ammonia and disease course. More observational, mechanistic and interventional data in humans are needed to move the field forward. We need systematic longitudinal descriptions of ammonia together with NAFLD disease stages and markers of metabolic and inflammatory disease activity. Ideally, such data should be accessible from the numerous randomised trials conducted. Regarding interventional studies, drugs with a supposed or demonstrated ammonia lowering mode of action are available or under investigation.

Another limitation is that hyperammonaemia may not be straightforward to identify. Blood sample analyses are notoriously sensitive to disturbances and particular care and diligence are required throughout collection, transportation and analysis, which poses challenges in clinical settings and even in clinical research protocols. This is particularly the case for moderate hyperammonaemia because local laboratories may calibrate their ammonia analysis for differential diagnostic purposes such as serious brain symptoms. Thus, there is no universal upper threshold for normal blood ammonia concentration or pathological hyperammonaemia in venous or arterial blood and we have to rely on local norms and criteria, and information on how these are defined. A way to aim for standardisation could be to normalise ammonia levels to an upper limit of normal at the respective reference laboratory.

## Perspectives

### Urea cycle activity as a target for NAFLD treatment

The first rational approach to target the decreased urea cycle activity in NAFLD would be to manipulate (viz. increase) urea synthesis capacity. Increased protein intake over time markedly increases the urea cycle’s capacity to clear nitrogen, and this mechanism is intact although weakened in cirrhosis.^127^ It is not known if the effect is intact in fatty liver diseases. Still, the effects of a high protein diet might be worth studying in NAFLD. The effect of high protein on urea synthesis seems not to involve the action of glucagon. As glucagon is the most potent upregulator of urea synthesis,^37^ treatment with exogenous glucagon or glucagon secretagogues seems an obvious option. However, urea synthesis becomes unresponsive to glucagon in cirrhosis,^128^ a phenomenon that would also be expected to occur in NAFLD, with its high glucagon levels. Also, glucagon may add to the risk of developing diabetes in such patients.^129^ Currently, there is no established way to counteract the epigenetic effects on the urea cycle *in vivo*. Pharmacologically, non-selective beta blockers have been shown to increase urea synthesis capacity in healthy individuals and those with cirrhosis ^130^; however, their long-term effect on ammonia is not known. Patients with NAFLD with arterial or portal hypertension may benefit from beta-blockade and it remains to be studied if they gain additional metabolic benefit from the treatment. Zinc is an obligatory co-factor for the OTC urea gene and zinc supplementation improves urea synthesis capacity in cirrhosis^131^ but the effect of zinc in NAFLD remains unknown. At present it is not possible or feasible to normalise the decreased urea synthesis capacity in NAFLD.

### Ammonia as a target for NAFLD treatment

The non-adsorbable disaccharide lactulose and, more recently, the antibiotic rifaximin as an add-on, are used as standard of care for HE and hyperammonaemia, with the aim of lowering ammonia production in the gut.^132^ Branched-chain amino acids and LOLA (L-ornithine L-aspartate) have been used with varying success to increase the incorporation of ammonia into glutamine.^133^ In genetic urea cycle disorders, hyperammonaemia is treated with metabolic ammonia scavengers, such as benzoic acid and phenylbutyrate (pro-drug to phenylacetate).^134^ Benzoic acid is conjugated to glycine in the liver to form hippuric acid, which is then excreted in the urine. Other ammonia scavengers are currently under investigation for the treatment of hyperammonaemia related to cirrhosis and acute liver failure, such as ornithine phenylactetate (OP).^135^^,^^136^ Transamination of ornithine produces glutamate which then binds to ammonia to form glutamine. Glutamine then binds to phenylacetate producing phenylacetylglutamine, which is excreted in the urine and removes two nitrogen atoms from the body. In bile duct-ligated rats, a reduction in blood ammonia levels using OP was observed^137^ and in a follow-up study this was associated with a reduction in HSC activation markers and portal pressure.^23^ Recently, in patients hospitalised with an episode of HE, OP dose-dependently reduced ammonia levels and an association between reduced ammonia levels and improvement in HE stage was observed.^138^ Ammonia lowering likely has a clinically meaningful beneficial effect on liver function and portal pressure in human cirrhosis, as indirectly demonstrated in clinical trials assessing lactulose for the prevention and treatment of HE, which showed reduced occurrence of decompensation episodes and reduced mortality.^139^ However, no clinical studies have investigated the direct effect of ammonia lowering on liver function and disease course.

Ammonia-lowering as a treatment for NAFLD has been investigated in *in vitro* studies and in an animal model. In steatotic rat liver slices, we demonstrated that adding OP to the medium decreased ammonia levels and reduced the severity of fibrosis, demonstrated by decreased collagen deposition.^47^ Also *in vivo*, we found that the activation of HSCs and subsequent fibrosis due to NAFLD-related reduced urea synthesis and hyperammonaemia can be reversed by ammonia scavenging using OP. In the same model, OP was reported to prevent hepatocyte cell death and reduce fibrosis – this was associated with the restoration of urea cycle enzyme gene expression and activity, and reduced ammonia concentrations as well as inflammation markers in liver tissue.^47^ Another study in a NAFLD mouse model used LOLA as an ammonia-lowering strategy, which had a beneficial effect on skeletal muscle, but failed to reduce ammonia levels, which was probably the explanation for the lack of improvement on liver histology.^49^

## Conclusions

As presented, there is now substantial circumstantial evidence for the concept that reduced urea synthesis caused by steatosis-induced dysfunction of urea cycle enzyme genes leads to hyperammonaemia, which contributes to the symptoms of NAFLD and is of pathogenic importance for its progression. The hypothesis incriminates ammonia as the effector and provides the rationale for its therapeutic targeting in NAFLD.

## Financial support

This research was generously supported by grants from The Foundation of Manufacturer Vilhelm Pedersen and Wife, and the 10.13039/501100009708Novo Nordisk Foundation (NFF19OC0055039).

## Authors’ contributions

KLT, RJ and HV conceived the study. KLT, PLE, AJCK and FDC drafted the manuscript. PLE and AJCK designed the figures. RJ and HV revised the manuscript. All authors read and approved the final manuscript.

## Conflicts of interest

Rajiv Jalan is the inventor of OPA, which has been patented by UCL and licensed to Mallinckrodt Pharma. He is also the founder of Yaqrit Discovery, a spin out company from University College London, Hepyx Limited and Cyberliver. He had research collaborations with Yaqrit Discovery. All other authors have nothing to disclose.

Please refer to the accompanying ICMJE disclosure forms for further details.
